# A novel method for dendrochronology of large historical wooden objects using line trajectory X-ray tomography

**DOI:** 10.1038/s41598-021-90135-4

**Published:** 2021-05-26

**Authors:** Francien G. Bossema, Marta Domínguez-Delmás, Willem Jan Palenstijn, Alexander Kostenko, Jan Dorscheid, Sophia Bethany Coban, Erma Hermens, K. Joost Batenburg

**Affiliations:** 1 Computational Imaging, Centrum Wiskunde & Informatica, Science Park 123, 1098 XG Amsterdam, The Netherlands; 2 Conservation and Science Department, Rijksmuseum, Hobbemastraat 22, 1071 ZC Amsterdam, The Netherlands; 3 Department of History of Art, University of Amsterdam, BG2 Turfdraagsterpad 15-17, 1012 XT Amsterdam, The Netherlands; 4 Leiden Institute of Advanced Computer Science, Leiden University, Niels Bohrweg 1, 2333 CA Leiden, The Netherlands

**Keywords:** Computational science, Scientific data, Imaging techniques

## Abstract

Dendrochronology is an essential tool to determine the date and provenance of wood from historical art objects. As standard methods to access the tree rings are invasive, X-ray computed tomography (CT) has been proposed for non-invasive dendrochronological investigation. While traditional CT can provide clear images of the inner structure of wooden objects, it requires their full rotation, imposing strong limitations on the size of the object. These limitations have previously encouraged investigations into alternative acquisition trajectories, including trajectories with only linear movement. In this paper, we use such a line-trajectory (LT) X-ray tomography technique to retrieve tree-ring patterns from large wooden objects. We demonstrate that by moving a wooden artifact sideways between the static X-ray source and the detector during acquisition, sharp reconstruction images of the tree rings can be produced. We validate this technique using computer simulations and two wooden test planks, and demonstrate it on a large iconic chest from the Rijksmuseum collection (Amsterdam, The Netherlands). The LT scanning method can be easily implemented in standard X-ray imaging units available at museum research facilities. Therefore, this scanning technique represents a major step towards the standard implementation of non-invasive dendrochronology on large wooden cultural heritage objects.

## Introduction

Scientific investigations of cultural heritage objects made from different materials play an important role in understanding the manufacturing process, establishing their chronology, attributing objects to artists, and deciding on conservation methods^1–5^. Because of the uniqueness of these historical objects, investigations requiring invasive methods (those that involve removing a sample from the object to be analysed either by destructive or non-destructive techniques) are carefully weighed against the potential knowledge gain^1,6^. Therefore, the demand for non-invasive methods has increased over the past years, and novel techniques are being developed^7–13^. Amongst these, X-ray-based imaging modalities such as X-ray fluorescence (XRF) spectroscopy^14^, radiography^15^ and CT imaging^16–18^ are important tools for investigating the chemical composition and the interior structure of art objects. Here, we focus on non-invasive X-ray absorption imaging for the purpose of tree-ring dating as a means to aid in the authentication of art-historical wooden objects.

Dendrochronology (tree-ring science) is the most exact method to date wood of (pre)historical objects, structures and artifacts^19,20^. Ring-width series in living trees can be merged to develop reference chronologies anchored in time, which can be used to identify the unique chronological position of tree rings in wood from (pre)historical contexts^21,22^. This is known as crossdating, whereby each ring is assigned an exact calendar year^19,21^. In traditional dendrochronology tree rings are measured in the transverse section of the wood. This requires a sample to be extracted from the object and placed under a microscope, or the growth pattern to be accessible in a cross-section so that it can be photographed and measured on the digital photos. Usually, optimal visualisation of the tree rings is only obtained after cleaning the surface with some abrasive method^6^. Such methods range from non-invasive simple brushing to highly-invasive procedures such as sand-blasting or even scraping with scalpel blades. The use of invasive methods is undesirable because they irreversibly alter the object. Furthermore, even when tree-ring patterns are accessible and the measurements can be done without invasive procedures, the longest tree-ring series may not be accessible from the outside. Obtaining the longest possible tree-ring series is crucial to the success of dendrochronological research, as longer series have higher chances to be dated by finding a statistical and visual match with the reference chronologies in a unique position (i.e. in one calendar year)^23^. The shorter the series, the higher the likelihood to obtain spurious matches in random positions, which precludes the possibility to identify which one is the correct date^19^. These considerations have prompted in recent years an increasing demand for the implementation of dendrochronology through non-invasive techniques.

The use of computed tomography (CT) for non-invasive imaging of tree rings has grown over the last decade, as it provides access to the inner structure of the wood when the tree-ring patterns cannot be retrieved by direct inspection on the surface^24–27^. The 3D information obtained by CT of the internal structure of the object has a decided advantage over 2D images, such as radiographs, yielding additional insights in e.g. the conservation state of a wooden object and previous restoration interventions^17,28–31^. Successful investigations using CT scanners at medical facilities, laboratories and synchrotron facilities have been performed on wooden objects of varying sizes such as historical instruments^28,32–34^, painted panels^25,35^, archaeological objects^36^ and sculptures^30^. However, the method is in general not well-suited for large objects. A full rotation is needed within the space between source and detector or the source and detector around the object, posing size constraints on the object. Moreover, the size of the detector determines the portion of the object that can stay in the field of view and thus can be imaged during one rotation. In addition, the size of the detector is usually linked to the detector pixel size and therefore a larger detector implies lower spatial resolution. To image large objects in laboratory setups, several CT scans have to be made and merged, requiring large amounts of data and high scanning and computation times^37^. As a result of such constraints, few large wooden objects, such as panel paintings and sculptures, have been scanned using CT thus far^25,30^.

Here, we present a line trajectory (LT) scanning technique for a laboratory setup that is suited for large objects such as large panel paintings, chests and cabinets. Rather than rotating the object (as is customary in CT), we move it on a linear trajectory between the X-ray source and detector, while recording radiographs. Scanning geometries with linear movement have been previously investigated^38–41^. Line trajectories in various configurations with one or multiple source-detector pairs have been used for security inspections^39^, knot detection in logs^42^ and medical applications^43^. Simulations of a LT scan show promising results for imaging features with a planar shape^44^. For rectangular, flat objects, computed laminography is another common method, usually requiring a full rotation of the object or source-detector pair with a tilted rotation axis^10,45–47^.

The novelty of the proposed LT scanning technique lies in exploiting the direct link between the features to be imaged and the scanning trajectory. We make use of the specific requirement for dendrochronology, providing a cut through the wood that shows the tree-ring pattern in the transverse section. In the following, we introduce the principles of this method and validate it with simulated data and test objects, discussing possibilities and limitations. Subsequently, a successful case study of an iconic wooden chest from the Rijksmuseum in Amsterdam (The Netherlands) is presented.

## Methods

### Dendrochronology and CT

**Figure 1:**
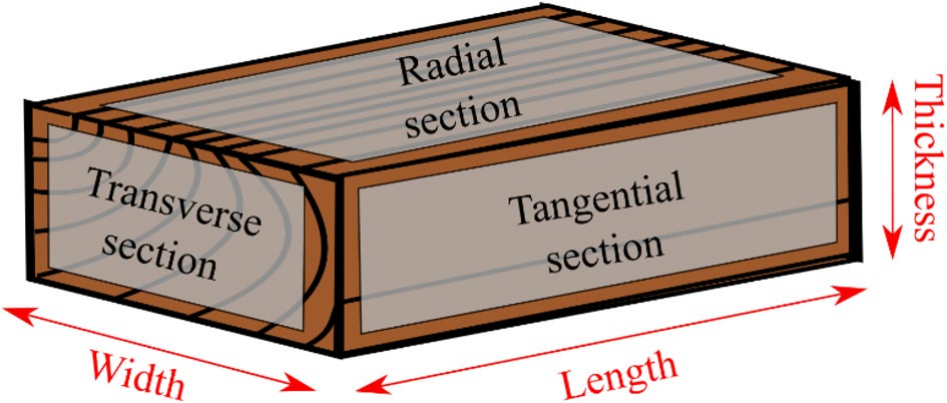
Schematic figure to illustrate the dendrochronological concepts of transverse, radial and tangential section and the definitions of length, width and thickness as used throughout this paper.

To date wooden objects or investigate their origin, the science of dendrochronology typically uses the relative width of tree rings. The tree rings are measured on the transverse section of the wood (Fig. 1) However, the transverse section is not always accessible on the outside of the object, for example due to how the plank was processed.

**Figure 2:**
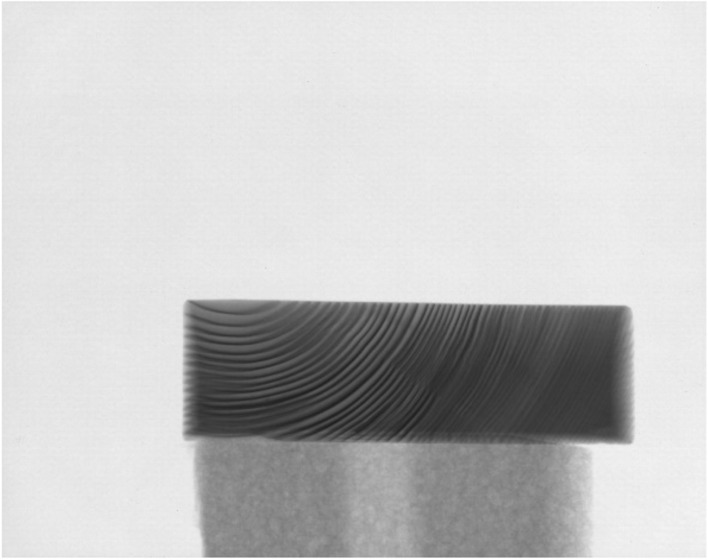
A radiograph of a wooden block, with the transverse section facing the source.

To overcome this limitation tree rings in a wooden object can be visualised using X-ray absorption imaging, because the differences in density within the wood provide contrast. The reason for this contrast is that more radiation travels through the less dense areas (earlywood) and less radiation through the denser areas (latewood). On a single radiograph, most rings will be projected at an angle, because of the cone shape of the X-ray beam. This means that on the radiograph denser and less dense areas are overlayed, blurring part of the rings. A single radiograph does therefore not suffice to extract the entire tree-ring pattern (Fig. 2).

During a regular CT scan, radiographs (or projections) are taken over a rotation circle of 360°. A 3D volume can be reconstructed from the 2D projections^48^. This reconstruction consists of 3D pixels, called voxels, that can be visualised as a stack of images (slices) at different depths in the object. For regular CT scans, reconstructions are commonly done using fast analytical algorithms^49^, but these are typically tailored to specific acquisition trajectories. Iterative tomographic reconstruction methods on the other hand, are slower but more generally applicable to acquisitions using non-standard trajectories^50^.

### Line trajectory scanning

**Figure 3:**
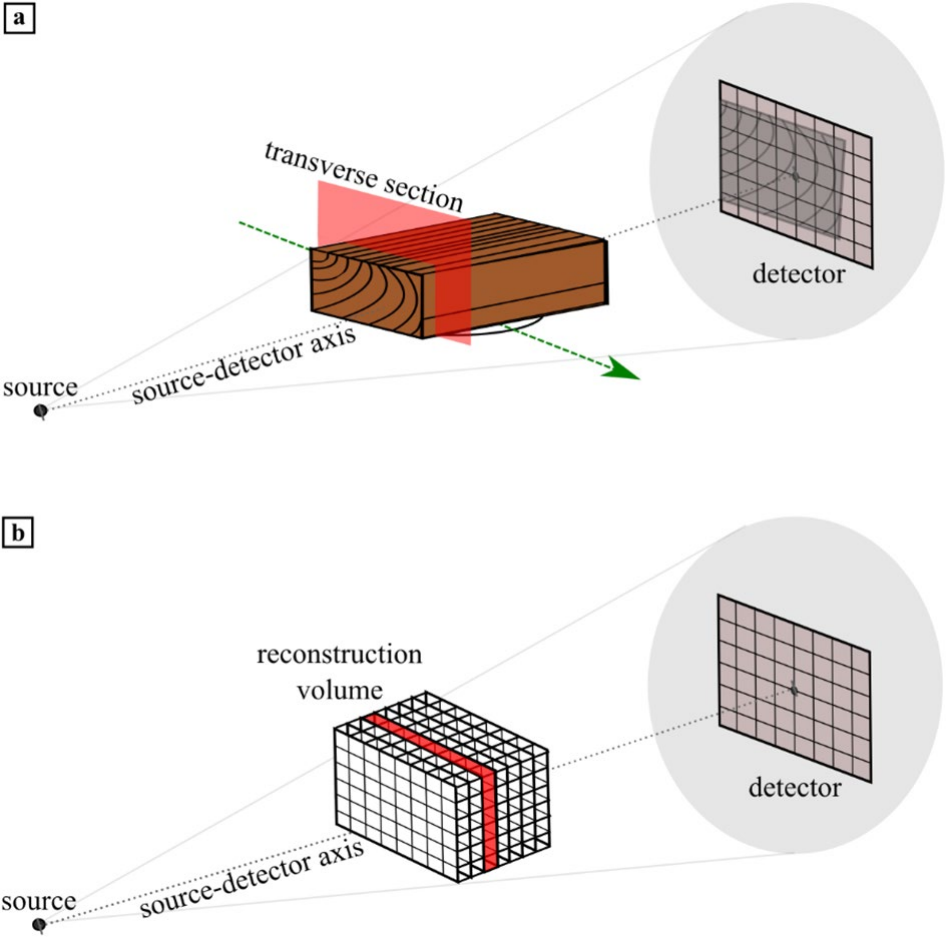
Schematic figures illustrating the LT scanning method. (**a**) Setup of the LT scan, where the object is placed with the transverse section perpendicular to the source-detector axis and is moved along the green arrow between source and detector. The transverse section is indicated by the red plane. (**b**) Schematic showing the location of the virtual reconstruction volume within the setup, a slice perpendicular to the source-detector axis is indicated in red.

The proposed scanning trajectory is a linear movement of the object between the source and detector (Fig. 3a). The internal features we want to capture, the tree rings, are curved surfaces along the longitudinal axis of the tree. As the data is not collected over a complete angular range, this will however not yield a perfect reconstruction. The reconstructed 3D image is expected to be smeared out along the source-detector axis. The tree ring surfaces are elongated in the direction of the longitudinal axis. If these are placed parallel to the source-detector axis, the typical smearing effect across slices is along the direction of the longitudinal axis. Although this smearing may have a large quantitative impact on the attenuation values in the reconstruction, due to the high similarity of wood structure in that direction, the influence of the smearing is effectively small on the slices perpendicular to the source-detector axis. This enables a sharp reconstruction of the tree-ring pattern of the transverse section in those slices. If the pith lies outside the plank, the tree ring surfaces are mainly vertically oriented. Images are then taken along and at slight angles of these surfaces. We expect that this will lead to the sharp imaging of the tree rings. If the orientation of the longitudinal axis is sufficiently aligned with the source-detector axis, the desired transverse section lies in a reconstruction slice perpendicular to the source-detector axis (Fig. 3). Slices in this direction should then provide images of the transverse section suitable for tree ring acquisition.

### 3D reconstruction and slice selection

In this paper, we use the simultaneous iterative reconstruction algorithm (SIRT)^50^ to obtain reconstructions, because of its suitability to deal with non-standard trajectories. This reconstruction algorithm transforms the data into a high dimensional system of linear equations, which is then solved by iteratively updating the reconstruction based on the difference between a forward projection of the current estimate and the acquired data. A possible drawback of the SIRT algorithm is the required computation time and memory. This can partly be overcome by reducing the number of voxels in the reconstruction volume. When the scanned objects are long, this can be achieved by extending the voxels in the length direction, i.e. producing cuboid voxels. The effect on the quality of the transverse slices is negligible because of the high similarity of the wood structure at the scale of the voxels in the direction of the longitudinal axis.

From the reconstructed volume, slices need to be selected for dendrochronological analysis. As the side of the object closest to the source has the highest magnification factor on the detector images, this will have the best image resolution in the reconstruction. The voxel size is therefore chosen based on magnification of the front of the object. Due to the previously described smearing effect, the exact front of the object does not yield the sharpest slice. Based on inspections of results, we choose to investigate slices around 25% from the front of the object. As the features have a strong similarity in the scanning direction, the measurements will be comparable across slices that are close to each other. The measurement of tree rings is a manual process, in which the expert knowledge of a dendrochronologist is required. Through the inspection of images together with a dendrochronologist, slices that would yield the most measurable images (i.e. showing the sharpest tree rings) were selected. Measurements can be performed either on one slice or on multiple slices and averaged subsequently to compensate for slight variation in manual measurements.

### Scanning procedure and analysis of the wooden test objects

The scans of two wooden test objects were carried out at the FleX-ray laboratory^51^ of the Centrum Wiskunde & Informatica (CWI) in Amsterdam (The Netherlands). This scanner in laboratory setup allows for flexible adjustment of the components and is therefore well suited to investigate new scanning trajectories. It can image objects up to approximately 50 cm × 50 cm × 50 cm.

The wooden test objects were placed on the sample stage, aligning their centre with the centre of the stage. We chose the range of translation as far to the left and right as possible within the space constraints. We determined the source power, energy and exposure time to gain sufficient contrast of the early- and latewood of the tree rings by visually inspecting the projections. Then, we calculated the distance that the object moved between projections, ensuring that a point on the front of the object would move less than one detector pixel in between two projections. Taking a high number of images should provide better reconstruction quality; therefore it is recommended to move the object at short intervals along the trajectory to increase the number of projections. To process the scan, the projection data were first flat- and darkfield corrected. For all the reconstructions the Flexbox software^52^ was used, with reconstruction algorithm SIRT. From the reconstruction, one or more slices were selected for measurement.

The transverse surface of both test planks was moreover prepared for dendrochronological research cleaning with sharp scalpel blades. Chalk powder was applied to the surface to enhance the visualisation of the tree-ring boundaries^19,53^. The surface was photographed with a compact digital camera on macro mode to proceed with the measurement of the tree rings.

Tree-ring widths were measured on the photographs and selected slices from the reconstruction by manually placing the coordinate points on the ring boundaries following a reference line perpendicular to the boundaries using the software package CooRecorder & Cdendro^54^. CooRecorder registers the distance between two points (which are equivalent in this case to the ring width) as coordinates, and CDendro converts the coordinates into metric units, creating a series of ring widths (commonly known as tree-ring series). Next, we used PAST4 v.4.3^55^ to crossdate the tree-rings series. This software automatically compares pairs of tree-ring series while performing several statistical tests for each overlapping position. To measure the goodness-of-fit we considered in PAST4 the Student’s *t* test calculated after normalisation of the data as described by Baillie and Pilcher^56^ (TBP), the percentage of parallel variation, or ‘Gleichläufigkeit’^57^ (Gl), which is a non-parametric test that reflects the synchronicity between overlapping portions of tree-ring series, and the significance level of the %PV. The Student’s *t* test is based on the correlation coefficient (*r*). Identical tree-ring series would yield TBP = 100 (resulting from a *r* = 1) and a Gl = 100% regardless of the length of the overlap. Tree-ring series obtained from the same sample should be very similar, although never identical due to differences in the manual placement of coordinates points. Still, they should yield high TBP and Gl values (e.g. TBP > 10 and Gl > 75%) even for short overlaps.

## Experiments and results

### Test case 1: small conifer block

**Figure 4:**
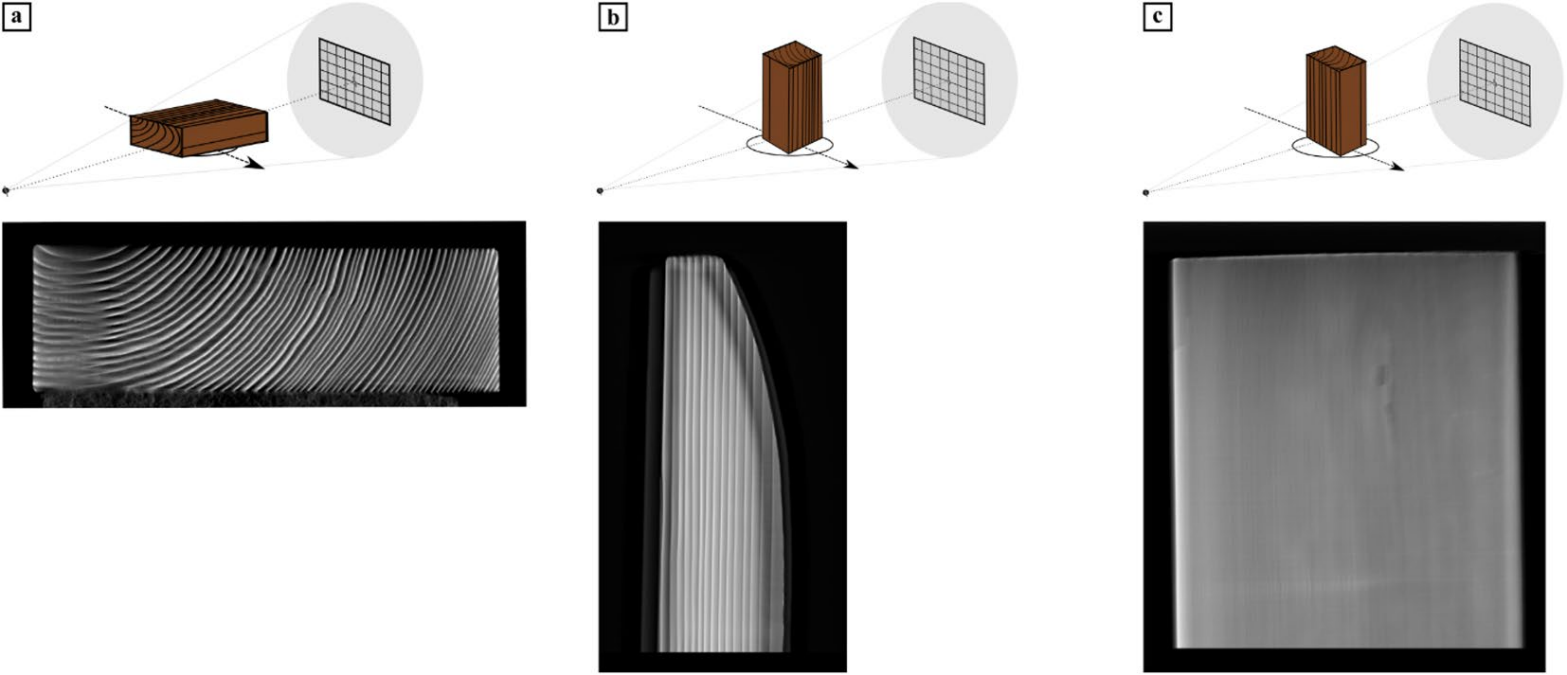
A reconstruction slice (bottom row) perpendicular to the source-detector axis from three LT scans. The wooden block was placed in three different orientations shown on the top row: the transverse section (**a**) perpendicular to the source-detector axis; (**b**) perpendicular to the vertical axis, width aligned with the source-detector axis; and (**c**) perpendicular to the vertical axis, width perpendicular to the source-detector axis. Only (**a**) is useful for dendrochronological investigation.

A small wooden block of conifer wood (3 cm thickness × 10 cm width × 12 cm length) was first chosen to assess how the positioning of the object affects the reconstruction. For this, we took three LT scans with the wooden object in different orientations^58^. For the LT scans 2,201 projections over translation range (− 110 mm, 110 mm) (Fig. 4a,c) and 2001 (Fig. 4b) projections over translation range (− 100 mm, 100 mm) were taken at tube settings 90 kV and 40 W. The reconstructions were performed with 100 iterations of SIRT and voxel size 66.9 micron. The results (Fig. 4) clearly show that the LT scanning technique only yields images suitable for dendrochronological measurements, when the transverse section is facing the source (Fig. 4a) and the tree rings are thus parallel to the source-detector axis. As the other orientations do not provide measurable images, the question arises how a slight tilt of the tree rings with respect to the source-detector axis affects the measurability. In the next section, we will show simulated results to investigate the effect of tree ring tilt.

**Figure 5:**
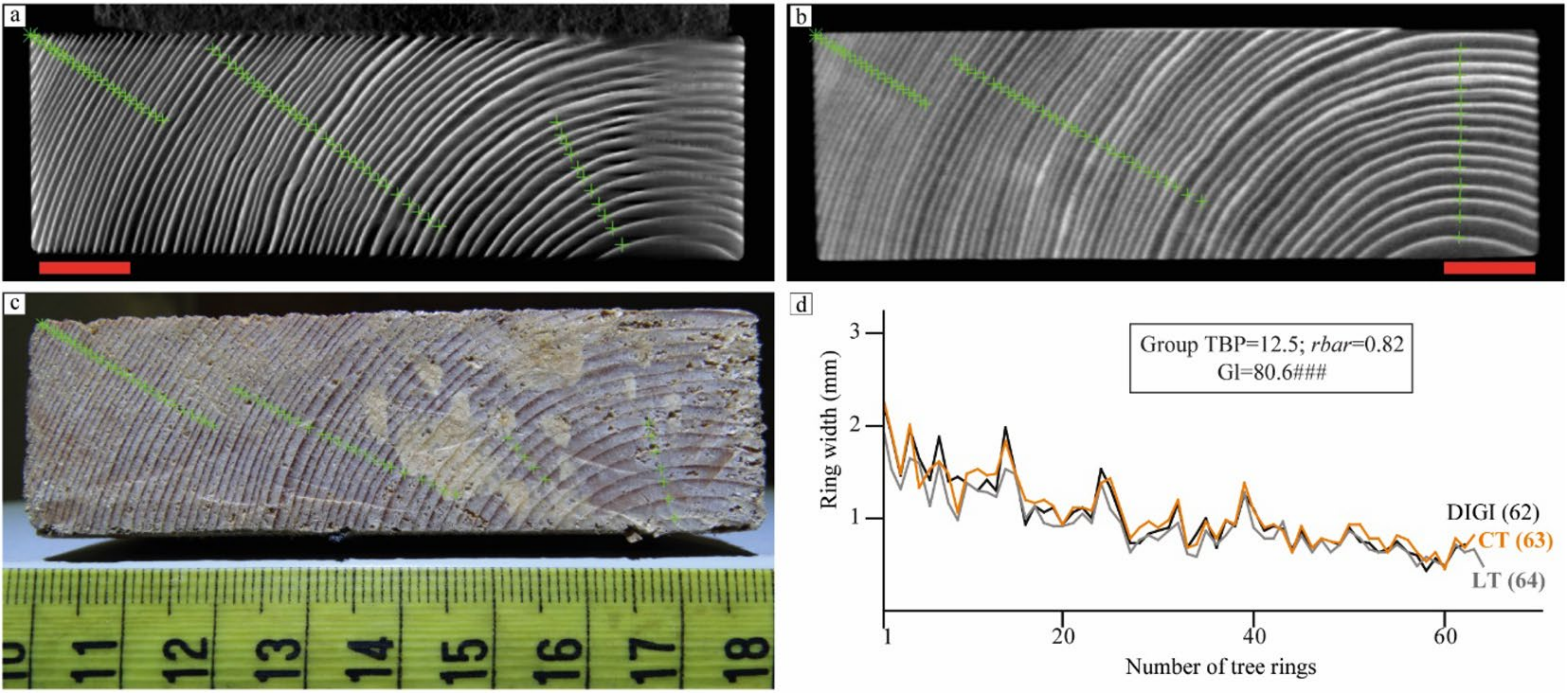
Results of tree-ring measurements on Test plank 1, crosses are placed at the border between rings. Red scale bars indicate 1 cm. (**a**) LT reconstruction, voxel size 66.9 micron. (**b**) CT reconstruction, voxel size 66.9 micron. (**c**) Digital photograph. (**d**) Visual and statistical crossdating between the measurements obtained from the different images. All three measurements show high similarity in pattern and magnitude (manual measuring and measured paths account for the slight differences). TBP, Student’s t-value as implemented by Baillie and Pilcher^56^ for tree-ring studies; rbar, mean correlation coefficient; Gl, mean percentage of parallel variation between the overlapping portion of the compared tree-ring series^57^ accompanied by its signification level (^###^p < 0.0001).

The size of the first test object allowed a full CT scan to be made to validate that the ring-width pattern measured in a LT scan (Fig. 5a), a full CT reconstruction (Fig. 5b), and a digital photograph (Fig. 5c) would yield the same tree-ring measurements. Therefore, a CT scan^59^ and a digital photograph were also recorded of this object. For the CT scan 2915 projections over a full rotation were recorded at 90 kV and 30 W. Similar as for the LT scan, the reconstruction was performed with 100 iterations of SIRT and the voxel size was 66.9 micron for both scans. The object was positioned on the source-detector axis in such a way to allow for a full CT scan while remaining within the field of view of the detector for 360°.

The results show that the measurements are indeed comparable in pattern and magnitude (Fig. 5d), and the observed differences can be attributed to different paths of the measurements and inaccuracies related to manual measuring. We observe that the horizontally oriented parts of the tree rings closer to the pith are blurred in the LT image but that this does not hinder the measurement as that is taken perpendicular to the tree-ring boundaries. Both reconstructions provide a good measurement match with the gold standard measurement on the digital photograph, which shows that the results from both reconstruction images are suitable for dendrochronological investigation.

### Test case 2: Simulations

**Figure 6:**
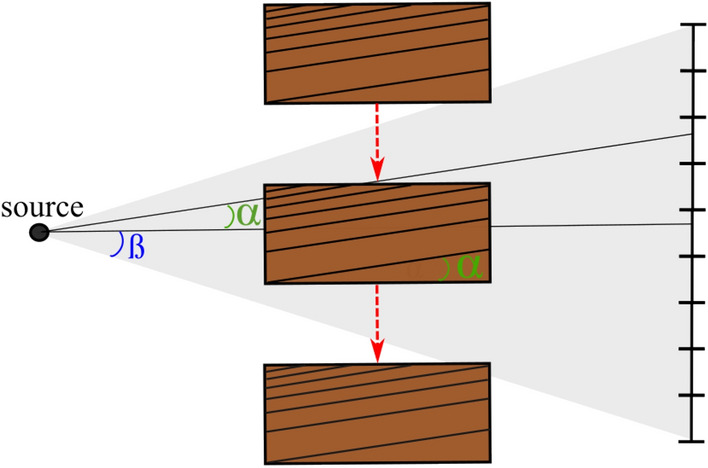
Schematic representation (seen from above) of the LT scan, with tree-ring tilt α and cone angle ß.

In practice, it is not feasible to have the tree rings exactly aligned with the source-detector axis. A tree-ring tilt is common and can be caused by the growth conditions of the tree, by how the wood has been processed (i.e. sawn at an angle) or by imperfect alignment of the wood in the scanner. The question therefore arises what the influence is of a small tree ring tilt on the performance of the LT method. To answer this question, we performed a small simulation study. The tree-ring tilt (α in Fig. 6) is 0°, when the longitudinal growth direction of the tree rings is parallel with the source-detector axis (α = 0°). The X-rays in the centre of the beam are then parallel to the rings. The cone-beam X-ray source emits rays in an angular range of ß degrees (see ß in Fig. 6). For tree-ring tilt smaller than ß there are still rays that are parallel to the tree rings. Therefore, we expect the method to perform well if the tree-ring tilt is sufficiently smaller than the cone angle.

**Figure 7:**
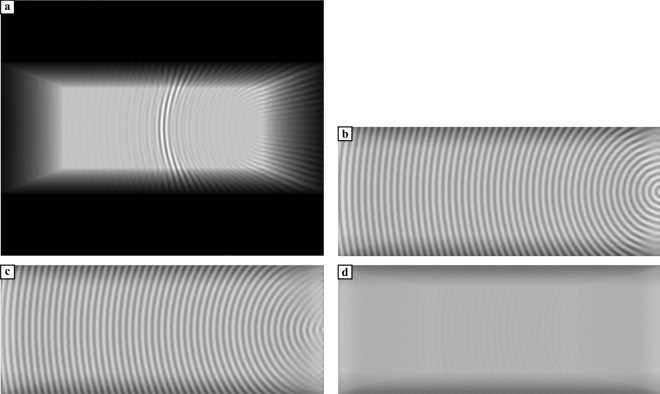
Simulated data and reconstructions. (**a**) Simulated projection. (**b**) Reconstruction with tree-ring tilt 0 degrees. (**c**) Reconstruction with tree-ring tilt 8 degrees. (**d**) Reconstruction with tree-ring tilt 15 degrees. Brightness and contrast settings are equal for all three reconstructions. The similarity between (**b**) and (**c**) demonstrates that the method is robust for small tilt in the tree rings.

We simulated a dataset in a best-case scenario in which the tree rings are perfectly concentric rings. The simulated datasets were created, with a tree-ring tilt α set at 0°, 8° and 15° and with a cone angle ß of 9°. The simulated data projections (Fig. 7a) were generated with the ASTRA-Toolbox^60^. Reconstructions were made using the SIRT algorithm with 50 iterations and a slice perpendicular to the source-detector axis at 25% of the length of the simulated object was selected. Note that on the projection (Fig. 7a) the tree rings parallel to the source-detector axis are sharp, whereas the other rings are blurred because the X-rays travel through the object at an angle, thus overlaying rings on the projection. Results show that for tree-ring tilts smaller than roughly 9°, the reconstructed images are similar (Figs. 7b,c). When the tree-ring tilt is higher, the image becomes blurred (Fig. 7d) and not suitable for tree-ring measurements. It must be noted that the cone beam angle ß is not a hard limit for the tree-ring tilt. A key conclusion of the simulations is that the LT method is robust to realistic tree-ring tilt. Moreover, in these simulated images we observe that the horizontally oriented part of the tree rings (bending around the pith) are slightly blurred, similar to the previously shown reconstruction of test object 1. This effect increases with the tree-ring tilt.

### Test case 3: conifer plank

**Figure 8:**
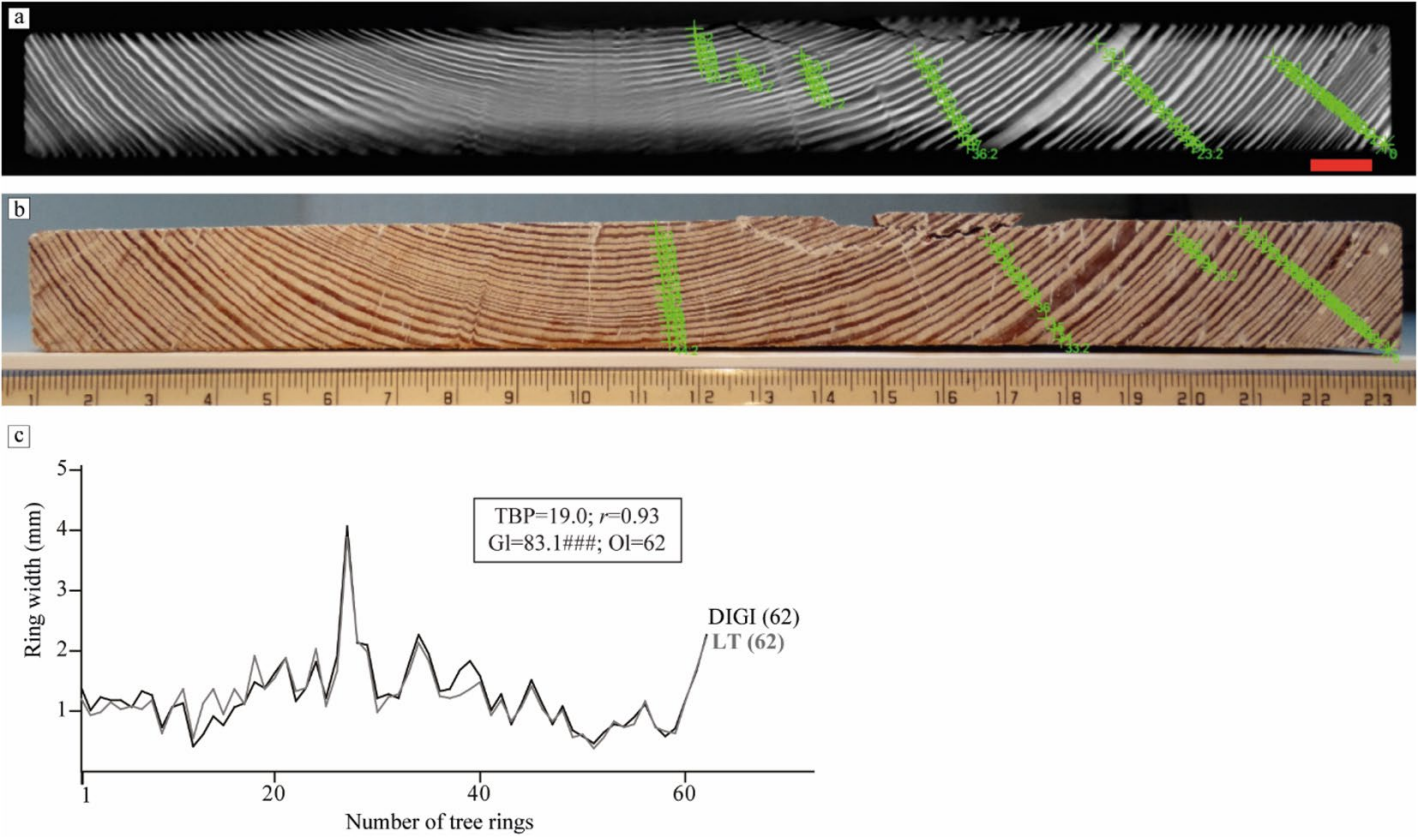
Dendrochronological results on the conifer plank. (**a**) LT reconstruction. Red scale bar indicates 1 cm. (**b**) Digital photograph. (**c**) Visual and statistical crossdating between the tree-ring series obtained from both images. The series have the same number of rings and show an outstanding synchronisation. TBP, Student’s t-value as implemented by^56^ for tree-ring studies; rbar, mean correlation coefficient; Gl, mean percentage of parallel variation between the overlapping portion of the compared tree-ring series^57^ accompanied by its signification level (^###^p < 0.0001).

A plank of conifer wood of 2 cm thickness × 23 cm width × 15 cm length, which was too large to be imaged in a single CT scan, was selected for the second test. It was scanned using the LT scanning technique with translation range (− 150 mm, 150 mm). The front of the object was placed as close to the source as possible, while ensuring that it still moved from outside the field of view of the detector on one side to outside the field of view on the other side. The scan took eight minutes and 1875 projections, with tube settings 70 kV and 30 W^61^. The reconstruction was performed with 400 iterations of SIRT and voxel size 20 micron, the voxels were elongated in the growth direction (1:1:4 scale) to reduce reconstruction time and memory. We inspected the slices at 25% and 30% of the object length and decided to use the slice at 30% for tree-ring measurements (Fig. 8a).

The preparation of the surface of the plank and the acquisition of the tree-ring widths on the digital photograph was done following the same procedures as in Test case 1 (Fig. 8b).

As expected from the simulations and the first test scans, the horizontally oriented tree rings appear blurred in the reconstructed image. Given that tree rings are measured along a path perpendicular to the pith this does not hinder the acquisition of tree-ring widths in that portion of the wood. The crossdating results show a good match between both tree-ring series, which is illustrated by an excellent visual match and high statistical values (Fig. 8c). This validates that the LT scanning method provides accurately measurable images for dendrochronological research. The narrowest ring in this plank is 0.34 mm wide and has been neatly captured in the LT image, proving that this method can be used successfully to retrieve tree-ring patterns of slow-grown trees, which were often used in the production of art works such as sculptures, furniture and panel paintings^62,63^.

### Case study: the book chest of Hugo de Groot

**Figure 9:**
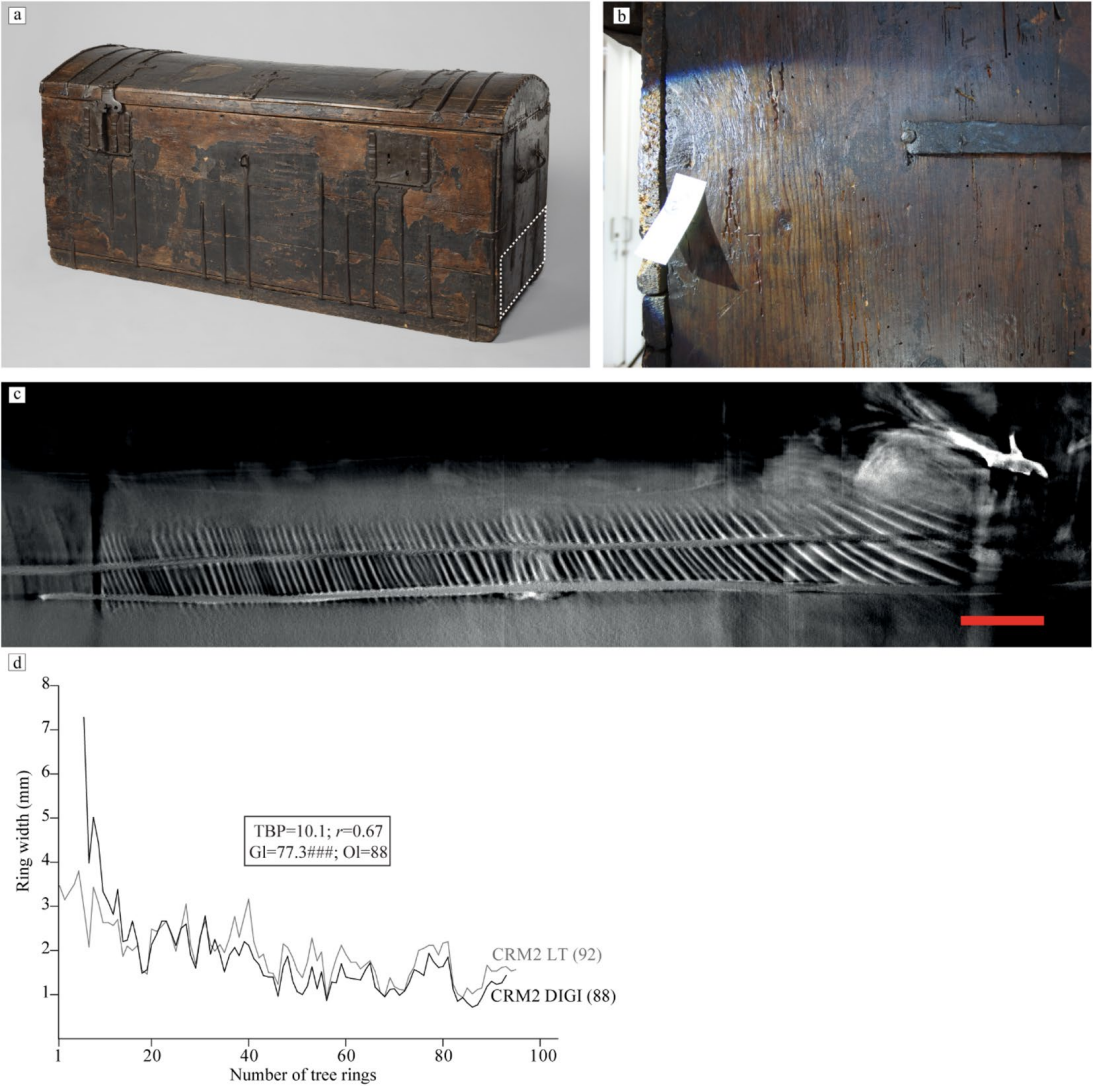
Results of the case study. (**a**) The Hugo de Groot book chest (NG-KOG-1208). The white rectangle indicates the lower plank of the right side of the chest, which was selected for this study. (**b**) Digital photograph on the radial/tangential section. (**c**) One of the LT reconstruction slices of the cross-section used to measure the tree rings, red scale bar indicates 1 cm. (**d**) Visual and statistical crossdating results between the tree-ring series obtained from the digital photographs (labelled CRM020_DIGI) and the one obtained from the LT reconstructed image. The series obtained from the digital image has seven rings less than the one from the LT reconstruction image and shows a slightly distorted pattern (magnitude-wise) due to measuring in the radial/tangential section of the wood.

Finally, we tested the LT scanning method on an iconic object from Dutch history at the Rijksmuseum collection: a book chest (object number: NG-KOG-1208) in which Hugo de Groot allegedly hid to escape imprisonment (Fig. 9a)^64^. Hugo de Groot (1583–1645) was a jurist and writer who was sentenced in 1619 to spend the rest of his life in prison in Loevenstein Castle, because of political disputes. He was allowed to write and receive books in large wooden chests, and in 1621, he performed a masterly escape from the castle hiding in one of the chests. The chest in question remained in his family for several decades afterwards, but its trail disappeared in the eighteenth century^65,66^. Three chests in Dutch museum collections were potentially the original chest in which Hugo de Groot escaped. Research into the origin of these was broadcast in the TV series 'Historisch Bewijs' (Historic Evidence)^66^. In the context of this investigation the opportunity arose to test the LT scanning technique on a large cultural heritage object.

The chest is large enough to have contained a person (73 cm width × 160 cm length × 75 cm height). These dimensions hamper scanning possibilities at most CT facilities, including the FleX-ray laboratory. However, the Rijksmuseum houses X-ray equipment within the building dedicated to Conservation and Science (the Ateliergebouw). The dimensions of the chest were too large to rotate it fully, a CT scan was therefore not possible. We could however carry out an LT scan to validate our technique.

LT scanning was carried out with tube settings 150 kV and 525 W and 1325 projections were recorded^67^. A ‘ruler’ with metal nails inserted every centimetre was used to register the translation between recorded projections. The magnification was then estimated based on the distance between two nails and the translations using cross correlation of the nail locations^68^. By this approach the accuracy of the experimental parameters is limited, but as demonstrated by the experimental results this does not compromise the robustness of the method. The reconstruction was performed with 100 iterations of SIRT and voxel size 90 micron.

A plank whose transverse edge was not accessible for dendrochronology was selected for the study as an example of a scenario in which dendrochronology could not be carried out on the desired section. Digital photographs of the tree rings were however taken with a compact camera on macro mode from the radial/tangential section of the plank, which was visible on the outside of the chest (Fig. 9b). Due to the fact that the measurement cannot be performed perpendicular to the pith, but only at a slight angle (which becomes larger when closer to the pith), the magnitude of the tree-ring widths could be slightly distorted in the radial/tangential section. The growth pattern should however be the same as the one retrieved from the LT image (Fig. 9c) and could thus provide validation of the LT technique.

Apart from the expected differences in magnitude due to the measuring on the radial/tangential section, crossdating results show an excellent match between both tree-ring series (Fig. 9d). Furthermore, the reconstructed image allowed the visualisation and measurement of more rings on both ends of the plank, which were too distorted or not visible in the digital photos from the radial/tangential section. Consequently, the series obtained from the LT image contains a total of 92 rings, whereas the one from the digital photographs is shorter (88 rings). As the length of the tree ring series can be crucial to the dating of the object, obtaining a longer tree ring series is a valuable result. Although this plank remains undated, these results validate the LT method, and demonstrate that it provides high quality images from large objects for dendrochronological research.

## Discussion and conclusions

Our research demonstrates that the proposed LT X-ray tomography method provides reconstructed images suitable for dendrochronological research. We exploit the fact that only a slice (instead of a full 3D image) is needed for dendrochronology, and have validated and demonstrated the method by obtaining accurately measurable images on simulations, test objects and a large wooden chest from the Rijksmuseum collection. By moving the object on a linear trajectory between the source and detector, with the transverse section of the wood facing the source, sharp images have been obtained where tree rings as narrow as 0.34 mm were clearly discernible. The results from our experiments open the door to future investigations of a variety of wooden objects, ranging from large panel paintings and doors from cabinets to chests, tables and large sculptures.

The LT scanning method has many advantages for dendrochronology on large objects. First of all, there is no need for rotation of the object and only one scan is required, as opposed to CT, where a full rotation is required for multiple scans (tiles) if the object is larger than the detector frame. The method can therefore be implemented in systems with a small detector or a static setup, such as a C-arm, where source and detector cannot be moved independently from each other. This is the typical X-ray imaging setup present in museum research facilities (e.g. The British Museum, London^69^ and Rijksmuseum, Amsterdam). Secondly, LT scanning can be carried out relatively fast. A scan can be performed within the order of 10 min, considerably reducing the exposure time. CT scans can take several hours up to days depending on the number of tiles and radiographs and the illumination time per radiograph^37^. Thirdly, given that a specific section of the object is selected, structures within the object that are unsuitable for X-ray imaging or that reduce the image quality, such as metals, can be avoided. In CT scanning all those structures are captured in the image during the rotation, causing distortions in the reconstructed image. Another artefact that commonly occurs in CT scans is the so-called ring artefact. If the pith of the wood is aligned with the rotation axis of the CT scanner, these ring artefacts may distort the tree ring pattern. This is avoided in an LT scan because of the scanning direction along the longitudinal growth direction.

For the LT scanning method, it is important that the transverse section of the wood is as perpendicular as possible to the source-detector axis. Still, we have shown that measurable images can be achieved when this alignment is not perfect. This makes the method feasible for practical application, as perfect alignment can be difficult to attain due to the variable shape and direction of the tree rings.

We have illustrated that for each scanned object there were object-specific considerations. Every object is different and therefore it is not possible to give one general set of guidelines that will guarantee measurable results. Optimal tube and detector settings need to be investigated and alignment is different for each object. The acceptable radiation dose should also be considered for each object. The effect of high radiation dose on artworks has not been sufficiently investigated yet, and therefore further tests need to be implemented to understand the short- and long-term effect of X-rays on art-historical materials.

The scanning direction of the LT method can be a limitation. It is necessary to scan in the longitudinal direction of the tree rings, which is often in the length direction of the plank. The total amount of material may be too thick to obtain enough contrast to distinguish the tree rings. Even if contrast can be obtained, high energy and power will be necessary in most cases, increasing the radiation dose.

We moreover found that the metal parts in the chest caused lower reconstruction quality. In this case, the effect was not so severe as to obstruct the measurement of tree rings. This may however be the case for other art objects, as these often contain metal parts.

For the test objects in this paper the pith of the tree lies outside the sample. We have shown that the imaged tree rings become blurry with larger curvature. The method may therefore be less suited for planks with the pith in the sample. However, we expect that this limitation will not often hamper the dating of the sample in practice, because for a dendrochronological measurement one line perpendicular to the tree ring direction is measured and the younger tree rings further from the pith are the most important for obtaining a felling date.

For the practical application of the LT scanning method, the input of both dendrochronologists and imaging scientists is required. The reconstructions should be discussed to select the slice that is best suited to measure the tree rings. The accuracy of the results increases when multiple reconstructed images at different depths are used for tree-ring measurements. Although the reconstruction of large LT datasets imposes substantial computational demands, we foresee that faster reconstruction may be possible. Additional research is needed to optimise reconstruction time.

In conclusion, LT X-ray tomography caters to the increasing demand for non-invasive research methods providing a novel and powerful technique for dendrochronology of large objects, which due to their size would be deemed unsuitable for CT imaging.

## Data Availability

The datasets generated and analysed in the current study are available in Zenodo repositories. Test object 1 (small conifer block): 10.5281/zenodo.4533882 (CT) and 10.5281/zenodo.4541555 (LT). Test object 2 (conifer plank): 10.5281/zenodo.4533887. Case study (chest): 10.5281/zenodo.4533923.

## Code availability

The code used for generating simulated data can be found on Github: https://github.com/fgbossema/line_trajectory_simulations.
